# Integrated Phenotypic–Genotypic Analysis of *Latilactobacillus* *sakei* from Different Niches

**DOI:** 10.3390/foods10081717

**Published:** 2021-07-25

**Authors:** Ying Chen, Nan Li, Shenxi Zhao, Chuan Zhang, Nanzhen Qiao, Hui Duan, Yue Xiao, Bowen Yan, Jianxin Zhao, Fengwei Tian, Qixiao Zhai, Leilei Yu, Wei Chen

**Affiliations:** 1 State Key Laboratory of Food Science and Technology, School of Food Science and Technology, Jiangnan University, Wuxi 214122, China; cyingjiangnan@163.com (Y.C.); zsx291382609@163.com (S.Z.); 13132551195@163.com (C.Z.); duan_hui2008@126.com (H.D.); xiaoyue_jiangnan@163.com (Y.X.); yanbowen2011@foxmail.com (B.Y.); zhaojianxin@jiangnan.edu.cn (J.Z.); fwtian@jiangnan.edu.cn (F.T.); zhaiqixiao@jiangnan.edu.cn (Q.Z.); chenwei66@jiangnan.edu.cn (W.C.); 2 State Key Laboratory of Dairy Biotechnology, Shanghai Engineering Research Center of Dairy Biotechnology, Dairy Research Institute, Bright Dairy & Food Co., Ltd., Shanghai 200436, China; linan@brightdairy.com; 3 Department of Agricultural, Food and Nutritional Science, University of Alberta, Edmonton, AB T6G 2R3, Canada; nanzhen@ualberta.ca; 4 National Engineering Research Center for Functional Food, Jiangnan University, Wuxi 214122, China

**Keywords:** *Latilactobacillus sakei*, comparative genomics, carbohydrate utilization, antibiotic tolerance, CRISPR-Cas

## Abstract

Increasing attention has been paid to the potential probiotic effects of *Latilactobacillus sakei*. To explore the genetic diversity of *L. sakei*, 14 strains isolated from different niches (feces, fermented kimchi, and meat products) and 54 published strains were compared and analyzed. The results showed that the average genome size and GC content of *L.* *sakei* were 1.98 Mb and 41.22%, respectively. Its core genome mainly encodes translation and transcription, amino acid synthesis, glucose metabolism, and defense functions. *L.* *sakei* has open pan-genomic characteristics, and its pan-gene curve shows an upward trend. The genetic diversity of *L.* *sakei* is mainly reflected in carbohydrate utilization, antibiotic tolerance, and immune/competition-related factors, such as clustering regular interval short palindromic repeat sequence (CRISPR)–Cas. The CRISPR system is mainly IIA type, and a few are IIC types. This work provides a basis for the study of this species.

## 1. Introduction

*Latilactobacillus sakei* is a ubiquitous psychrophilic lactobacillus, which was first isolated from sake by KATAGIR et al. [1] in 1934. This species is a Gram-positive bacterium that exists in many niches, such as sour dough [2,3], fermented vegetables [4], fermented meat products [5], and human feces [6]. *L. sakei* has attracted the attention of researchers owing to its prominent bacteriocin-producing ability. The species can produce various class II bacteriocins such as Sakacin Q [7] and Sakacin P [8] and can inhibit a variety of food-borne pathogenic bacteria such as *Listeria monocytogenes* [9] and *Staphylococcus aureus* [10]. In addition, recent studies have shown that some antimicrobial proteins in *L. sakei* also act as inhibitors of foodborne pathogenic bacteria. Adriana Lopez-Arvizu et al. [11] found that the antimicrobial protein produced by *L. sakei* UAM-MG-3, had high homology with N-acetylmuramoyl-l-alanine amidase and was able to effectively inhibit the activity of *Listeria innocua,* promoting its application in food industry. Gao et al. [12] found that adding *L. sakei* C2 and its bacteriocin can inhibit pathogenic bacteria and prevent lipid oxidation of vacuum-packed cooked ham slices at cold storage temperatures. In addition, *L. sakei* is used as a starter for fermented meat products [13] and kimchi [14] in the food industry, ensuring product quality and accelerating the fermentation rate. It is worth noting that *L. sakei* also has a variety of probiotic functions, such as regulating immunity [15], improving metabolic syndrome [16], and relieving inflammatory diseases [17]. In recent years, some studies have shown that *L. sakei* can reduce the expression of pro-inflammatory cytokines related to psoriasis and improve the severity of psoriasis in mice [18]. Other research results show that *L. sakei* K040706 can enhance the phagocytosis of macrophages and increase the expression of immune regulators such as inducible nitric oxide synthase (iNOS) and cytokines, making it a candidate drug for immune stimulation [19].

In 2005, Chaillou et al. [20] sequenced the genome of *L. sakei* 23 K for the first time, which gives a foundation for the development of genome sequencing of *L. sakei*. With genome technology development, the genetic diversity and functional diversity of bacteria have been extensively studied. Chaillou et al. [21] revealed that *L. sakei* has three different phylogenetic lineages influenced by its different habitats. Nyquist et al. [22] analyzed the genome of *L. sakei* isolated from processed meat products, and found that the genes involved in nucleoside elimination, arginine catabolism, and coping with redox and oxygen level changes of these strains were all retained, which may be the key factors for their survival in meat products. Studies have shown that niches affect the genetic characteristics and evolution direction of specific species, and these strains show specific host adaptability in the evolution process. However, at present, the genome analysis of *L. sakei* is limited to a few isolated sources such as fermented meat products, and the gene analysis of *L. sakei* from feces and pickles is limited. To further explore the genetic information and evolution of *L. sakei*, more genomic analysis is necessary.

In this study, the genetic diversity of the core genome, pan-genome, carbohydrate utilization enzymes and CRISPR–Cas system of 54 publicly available genomes of *L. sakei* from the National Center for Biotechnology Information (NCBI) and 14 *L. sakei* isolated from human feces, fermented meat, and vegetables in this study, for a total of 68 *L. sake* strains, were analyzed using bioinformatics.

## 2. Materials and Methods

### 2.1. Isolation of Strains, Genome Sequencing, and Data Assembly

A total of 14 *L. sakei* strains were isolated from fecal samples, fermented meat products, and fermented vegetable products in different regions of China, as shown in Table 1. The strains were cultured in De Man, Rogosa, and Sharpe (MRS) medium [23] and incubated for 24 h at 37 °C. All the identified *L. sakei* strains using 16S rRNA sequencing were stored at −80 °C in 30% glycerol. Illumina Hiseq X Ten platform (Majorbio BioTech Co, Shanghai, China) was used to sequence the draft genomes of *L. sakei*, and 2 × 150 bp paired-end libraries and a paired-end library with an average read length of about 400 bp were constructed. SOAPdenovo was used to assemble the reads, and GapCloser software was used to fill the local inner gaps [24]. In addition, 54 publicly available genomes of *L. sakei* from NCBI (https://www.ncbi.nlm.nih.gov/, accessed on 30 March 2021) were used in this study.

### 2.2. Genome Features Prediction and Annotation

Glimmer 3.02 [39] (http://ccb.jhu.edu/software/glimmer/index.shtml, accessed on 5 April 2021) prediction software was used to predict the G+C content of each genome. The Swiss-Prot [40] and RefSeq non-redundant proteins (NR) databases [41] using Diamond software were used to annotate amino acid sequences, with an E-value of 1 × 10^−5^. Glimmer (http://ccb.jhu.edu/software/glimmer/index.shtml, accessed on 5 April 2021) can predict the coding sequence (CDS) of the genome.

### 2.3. Pan-Genome and Core-Genome Analysis

Using PGAP-1.2.1 software, genomes were examined based on protein sequences, annotation information, and nucleotide sequences, and then analyzed according to the Heap’s law pan-genome model [42], to calculate the pan-genome of *L. sakei*. The protein sequence alignment of 68 strains was completed using Orthomcl software, and a Venn diagram was constructed [43]. According to the clusters of orthologous groups (COG) (http://www.ncbi.nlm.nih.gov/COG/, accessed on 15 April 2021) assignments, the functions of the genome-encoded proteins were categorized.

### 2.4. Average Nucleotide Identity (ANI) Values and Phylogenetic Analyses

The method of calculating ANI was used to average the consistency of homologous genes of each pair for sequences [44]. The python script was used to calculate the ANI value between any two genomes (https://github.com/widdowquinn/pyani, accessed on 28 April 2021) [45], and the obtained matrix was clustered and visualized by R package heat map software.

Orthomcl v2.0.9 software was used to extract all orthologous protein sequences of 68 strains, and the homologous genes were clustered [46]. MEGA 7.0 software was used to construct the phylogenetic tree of the MAFFT-aligned sequence [47], which was modified by Evolgenius (https://evolgenius.info//evolview, accessed on 8 May 2021) [48].

### 2.5. Genotype/Phenotype Association Applied to Carbohydrate Metabolism

The annotation of carbohydrate utilization genes was performed using the Carbohydrate Active Enzyme Database (CAZy) [49]. Thereafter, the annotated results were analyzed by cluster analysis using HemI software [50].

The ability of 14 *L. sakei* strains to utilize 13 carbohydrates, D-cellose, soluble starch, glucose hydrochloride, saccharose, D-fructose, D-galactose, L-arabinose, D-mannose, D-xylose, D-maltose, L-sorbose, escalin, and D-trehalose, was determined. Carbohydrate stock solution at a concentration of 10 g/L was filtered through a 0.22 μm filter membrane, and then added to modified MRS medium (without carbohydrate). Thereafter, 0.5% (*w/v*) bromocresol purple solution was added as an indicator. *L. sakei* was cultured at 37 °C for 24 h with 1% inoculum in the culture medium, and the change in color was used to judge its utilization. The experiment was repeated three times.

### 2.6. Genotype/Phenotype Association Applied to Antibiotic Resistance

The predicted antibiotic resistance gene information in the genome was obtained by comparing the amino acid sequences of the strains with the comprehensive antibiotic research database (CARD, http://arpcard.mcmaster.ca, accessed on 24 May 2021) [51]. The strains were clustered using HemI software [50].

The microbroth dilution method was used to determine antibiotic resistance of *L. sakei* according to ISO 10932:2010 [52]. The following 11 antibiotics were detected: chloramphenicol, rifampicin, streptomycin, kanamycin, gentamycin, tetracycline, clindamycin, neomycin, erythromycin, ciprofloxacin, and vancomycin (all purchased from Sangon Biotech Co., Ltd., Shanghai, China). OD_625_ was determined using an enzyme-labeled instrument (Varioskan Lux, Thermo, Waltham, MA, USA) to determine the MIC of strain to antibiotics.

### 2.7. CRISPR Identification and Characterization of Isolated Strains

CRISPR loci, CRISPR repeats, and spacers in *L. sakei* were excavated and predicted using CRISPRFinder (https://crisprcas.i2bc.paris-saclay.fr/CrisprCasFinder/Index, accessed on 30 May 2021) [53]. The repeated secondary structure was predicted using RNAfold (http://rna.tbi.univie.ac.at/cgi-bin/RNAWebSuite/RNAfold.cgi, accessed on 30 May 2021) [54]. Phylogenetic analysis was performed based on amino acid sequence and CRISPR repeats of Cas1, Cas2, and Cas9 proteins. MEGA software (version 7.0; Sudhirkumar, PA, USA) was used to construct the phylogenetic tree.

## 3. Results

### 3.1. General Genome Characteristics of Latilactobacillus sakei

In our previous research, 14 *L. sakei* strains were isolated from different niches, such as human feces, fermented vegetable products, fermented meat products, and kimchi water (Table 1). Combined with the genome information of 54 *L. sakei* strains published in NCBI GenBank database, the genomes of 68 *L. sakei* strains were compared and analyzed (Table 1). The genome size of 68 *L. sakei* strains ranged from 1.54 Mb (*L. sakei* ERR260134-bin.14) to 2.19 Mb (*L. sakei* WiKim22) with an average size of 1.98 Mb. The average G+C content was 41.22%, ranging from 40.61% for *L. sakei* WiKim22 to 42.03% for *L. sakei* QAHLA3L8. The average predicted coding sequences (CDSs) of each genome numbered 1923, ranging from 1779 for *L. sakei* 23 K to 2142 for *L. sakei* FXJWS8M1.

### 3.2. Pan-Genome and Core Genes of Latilactobacillus sakei

**Figure 1:**
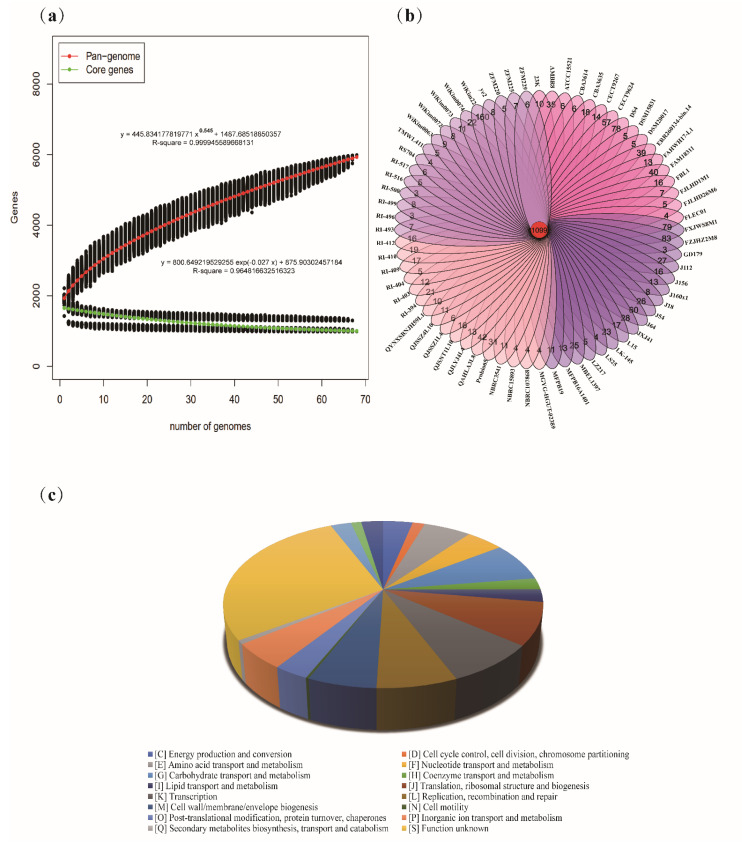
Pan and core genes of *Latilactobacillus sakei*. (**a**) Pan-genome and core genome, R stands for correlation coefficient; (**b**) Venn diagram displaying the unique and core genes; (**c**) Functional assignment of the core genome based on the COG database.

To study the genetic diversity of *L. sakei*, the pan-genome and core genes were analyzed. The functional relationship between the number of core genes and pan-genes and the number of sequencing strains was plotted (Figure 1a). The results showed that with an increase in the number of *L. sakei* strains, the number of pan-genes increased continuously, and the number of core genes tended to be stable. When the 68th strain was added, the number of ubiquitin genes was stable at 5983, and the number of core genes reached 993. The pan-genome curve shows an asymptotic trend, which may indicate that *L. sakei* has an open pan-genome. The specific core genes and homologous core genes of *L. sakei* strains were analyzed, and Wayne diagram was drawn (Figure 1b). Sixty-eight *L. sakei* strains had 1099 common core genes, and each strain had 4–160 unique core genes. Functional analysis of the core genes of *L. sakei* revealed that the core genome includes replication, transcription, translation, nucleotide metabolism, carbohydrate metabolism, amino acid metabolism, lipid metabolism, and other related genes. Among them, genes related to carbohydrate metabolism accounted for approximately 7.53% of core functional genes, 5.68% of core functional genes were related to amino acid metabolism; however, 28.24% of core genome functions are unknown (Figure 1c).

### 3.3. ANI and Phylogenetic Analyses of Latilactobacillus sakei

**Figure 2:**
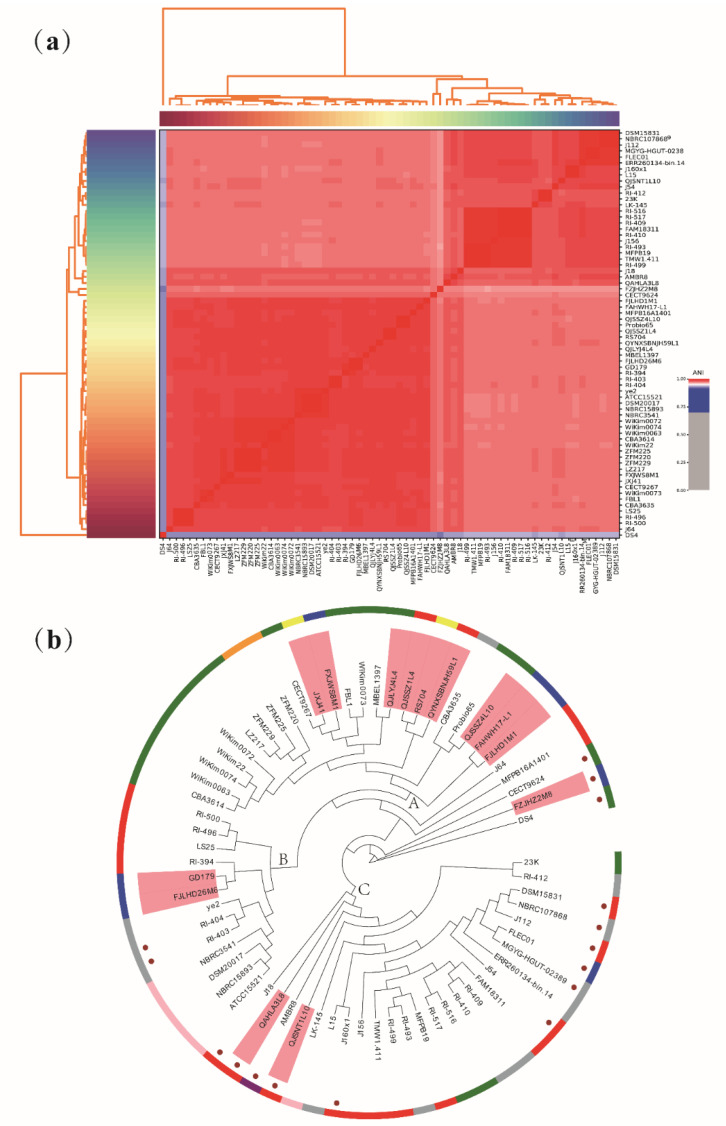
The Average Nucleotide Identity (ANI) and phylogenetic analysis of *Latilactobacillus sakei*. (**a**) Heatmap showing the ANI value among 68 *L. sakei* strains; (**b**) Phylogenetic tree based on orthologous genes. The salmon areas represent strains isolated in our laboratory. Brown circles represent strains with complete CRISPR–Cas system. The peripheral blue band represents strains from feces, the red band represents strains from fermented meat products, the green band represents strains from fermented vegetables, the pink band represents strains from fermented sake, the yellow band represents strains from kimchi water, the orange band represents strains from milk, and the source of gray band separation is unknown.

Average nucleotide identity (ANI) is a classic method that can analyze unique species or potential subspecies within the same strain. The generally accepted ANI boundary value is 95–96% [55]. When the ANI value is less than this boundary value, the strain may be a potential subspecies. Among the 68 *L. sakei* strains, except for *L. sakei* DS4, the ANI values of the other strains were higher than 97% (Figure 2a). The ANI value between strain DS4 and other *L. sakei* strains was only 92–93%, indicating that *L. sakei* DS4 may be a potential subspecies. However, this conjecture has not been confirmed by research, and whether *L. sakei* DS4 is a potential subspecies needs further discussion.

The phylogenetic tree was constructed based on homologous genes of 68 *L. sakei* strains to explore the phylogenetic relationships (Figure 2b). The phylogenetic tree was divided into two large clades, among which *L. sakei* DS4 was divided into one, which may be related to its low ANI value. The remaining strains were mainly divided into three evolutionary clades (A–C), and the strains isolated in our laboratory exist in all three evolutionary clades. In clade A, three strains were from feces, and the other six strains were from fermented products. Clade B was isolated from feces, whereas clade C was isolated from fermented meat products.

### 3.4. Genotype/Phenotype Association Analysis for Carbohydrates Utilization in Latilactobacillus sakei

**Figure 3:**
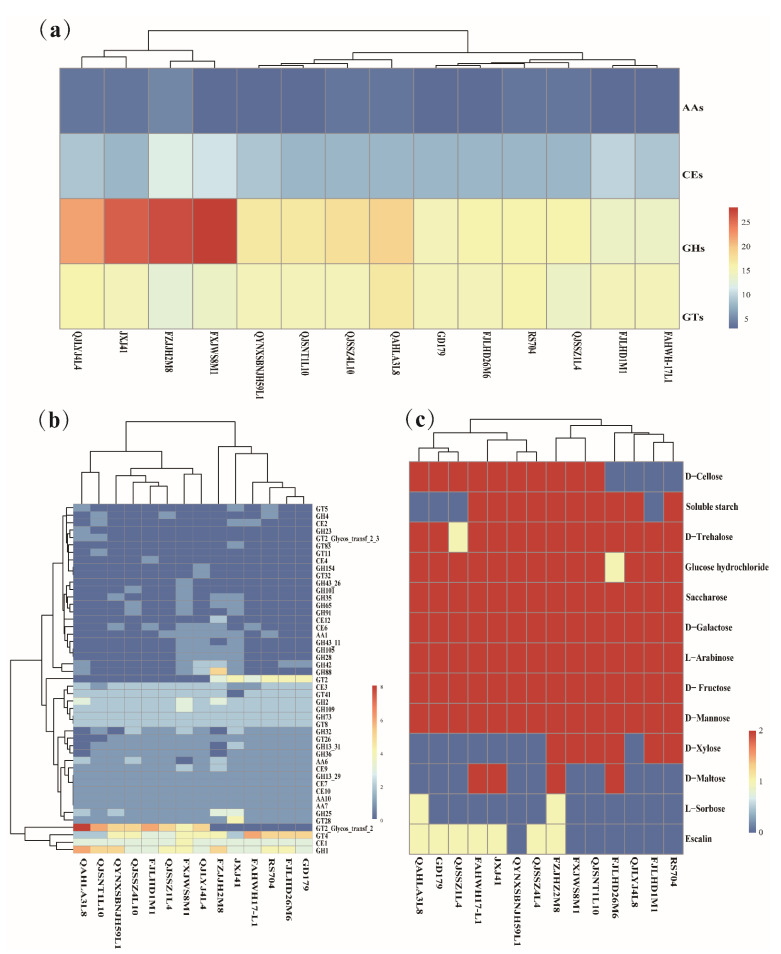
Genotype–phenotype analysis of carbohydrate utilization of *Latilactobacillus sakei*. (**a**) Comparative heat map of the number of genes in four families of carbohydrate active enzymes of different strains of *L. sakei*; (**b**) Thermographic analysis of carbohydrate enzyme activity-related genes of *L. sakei*; (**c**) Utilization ability of 13 carbohydrates by *L. sakei*.

To understand the carbohydrate utilization ability of *L. sakei*, the sequencing genomes of 14 *L. sakei* strains isolated in our laboratory were analyzed using the CAZy database. There are were carbohydrate-active enzyme families in 14 *L. sakei* strains, including auxiliary activity (AA) families, carbohydrate esterase (CE) families, glycoside hydrolase (GH) families, and glycosyltransferases (GTs) families. The GH family was the most abundant enzyme in *L. sakei*, followed by the GT family, and the number of carbohydrate-active enzyme genes encoding the CE and AA families decreased in turn (Figure 3a). Among these strains, AA10 (lytic cellulose monooxygenase (EC 1.14.99.56)), CE1 (feruloyl esterase (EC 3.1.1.73)), GH1 (β-glucosidase (EC 3.2.1.21)), GH2 (β-galactosidase (EC 3.2.1.23)), GH13_29 (α-amylase (EC 3.2.1.1)), GT4 (sucrose synthase (EC 2.4.1.13)), and another 16 active genes were found in all strains, whereas the distribution of the remaining genes was different in each strain (Figure 3b). For example, CE4 (acetyl xylan esterase (EC 3.1.1.72)) only existed in *L. sakei* FJLHD1M1, and AA6 (1,4-benzoquinone reductase (EC 1.6.5.6)) was absent in *L. sakei* FXJWS8M1. GH1, GH2, and GH109 accounted for a high proportion in the GH family, which are related to the catabolism of carbohydrates such as lactose and mannose. In addition, GT2 and GT4 accounted for a higher proportion in the GT family. Based on the clustering results of carbohydrate utilization active enzymes of *L. sakei*, it was found that there was no evident regularity between the clustering results of various strains and the host and geographical sources, indicating that there is diversity in the carbohydrate utilization ability of *L. sakei*.

To verify the genotype, the ability of *L. sakei* to utilize 13 carbohydrates was determined. All strains utilized glucose hydrochloride, D-mannose, D-fructose, L-arabinose, D-galactose, and sucrose, but had strain specificity for the utilization of D-maltose, D-xylose, soluble starch, L-sorbose, D-trehalose, D-cellobiose, and escin (Figure 3c). The metabolism of D-galactose is responsible for β-galactosidase, which belongs to GH2 and GH42 families. It is not difficult to find that all *L. sakei* strains containing GH2 or GH42 gene can utilize D-galactose. In addition, β-mannosidase in GH2 is responsible for mannose catabolism, promoting the utilization of mannose by 14 *L. sakei* strains. All strains contained GH13_29 family enzymes, which have α-amylase related to starch hydrolysis and trehalose-6-phosphate hydrolase related to trehalose hydrolysis. However, four strains did not show the ability to utilize soluble starch, and one strain showed the ability to utilize trehalose, which may be due to the lack of expression of GH13_29 in the four strains.

### 3.5. Genotype/Phenotype Association Analysis for Antibiotic Resistance in Latilactobacillus sakei

**Figure 4:**
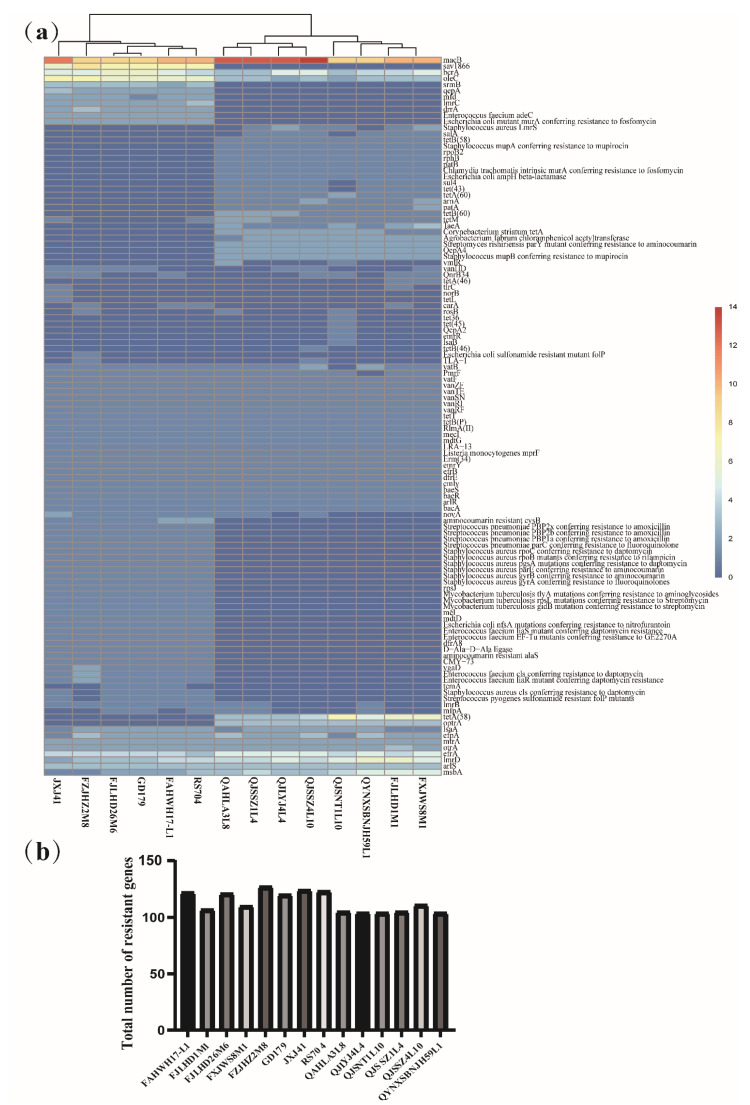
Genotype–phenotype analysis of antibiotic resistance of *Latilactobacillus sakei*. (**a**) Clustering heat map analysis of antibiotic resistance genes in *L. sakei*; (**b**) Total number of resistance genes of 14 *L. sakei* strains.

The genome of *L. sakei* was annotated using CRAD database. The annotation results showed that 117 resistance genes were predicted in 14 *L. sakei* strains (Figure 4a), including macrolide resistance genes (*macB*), glycopeptide resistance genes (*vanRF* and *vanRI*), aminoglycoside resistance genes (*baeR* and *baeS*), fluoroquinolone resistance genes (*mfd* and *mfpA*), lincoamide resistance genes (*lmrB* and *lmrD*), streptogramin resistance genes (*vatB* and *vatF*), tetracycline resistance genes (*emrY*, *rpsJ*, *tetM*, *tetB(P)*, and *tetT*), and rifamycin resistance genes (*rphB*). By comparing the total number of resistance genes of 14 *L. sakei* isolates from different sources, it was found that the resistance genes of *L. sakei* isolated from feces and kimchi water were higher than those isolated from fermented products (Figure 4b). Among them, the number of resistance genes of *L. sakei* FZJHZ2M8 was the highest (127), whereas that of *L. sakei* QJLYJ4L4 and *L. sakei* QYNXSBNJH59L1 was the least (104). In addition, the distribution of some resistance genes appears to be related to the isolated sources. For example, rifamycin resistance gene *rphB* and tetracycline resistance gene *tetB(60)* existed in all strains isolated from fermented products and a few strains isolated from feces. However, the resistance genes *dfrA8* and β-lactam *CMY-73* were mostly found in the strains isolated from feces and kimchi water; however, none of the strains isolated from fermented products contained these genes (Figure 4a).

Furthermore, the genetic traits of 14 *L. sakei* strains were matched, and the tolerance of *L. sakei* to 10 antibiotics such as erythromycin, vancomycin, streptomycin, kanamycin, gentamycin, neomycin, tetracycline, rifampicin, ciprofloxacin, and clindamycin was analyzed (Table 2). There were glycopeptide resistance genes and aminoglycoside resistance genes in all strains; therefore, all strains have good tolerance to typical glycopeptide antibiotics such as vancomycin and aminoglycoside antibiotics such as streptomycin, kanamycin, and gentamycin. Macrolide resistance genes were abundant in 14 strains of *L. sakei,* and the strains had good tolerance to erythromycin, except for *L. sakei* QJSNT1L10. *TetT* and *tetB(P)* genes related to tetracycline resistance existed in all strains; however, six strains were sensitive to tetracycline, which may indicate that *tetT* and *tetB(P)* are not the key genes of tetracycline resistance.

### 3.6. Prediction of CRISPR–Cas Systems in Latilactobacillus sakei

Fourteen *L. sakei* strains isolated from our laboratory were analyzed using the CRISPR–Cas system, and thirteen strains were identified as CRISPRs. However, only genomes with evidence levels above 1 were considered in this study due to differences in evidence level. Moreover, CRISPR, an orphan without Cas protein, was ignored because it could not silence foreign DNA. Two strains with complete CRISPR–Cas system were isolated from fermented products (QJSNT1L10 and QAHLA3L8), and one strain was isolated from baby feces (FZJHZ2M8), all of which belonged to class IIA or IIC, including Cas1, Cas2, Cas9, and Csn2 (Table 3). To further explore the distribution characteristics of the CRISPR–Cas system of *L. sakei*, the CRISPR–Cas system of 54 strains of *L. sakei* on NCBI was predicted. Among the 54 strains, 13 strains had complete CRISPR–Cas system, and these systems were all IIA or IIC. Most of these 14 strains were isolated from fermented products (Table 3).

**Figure 5:**
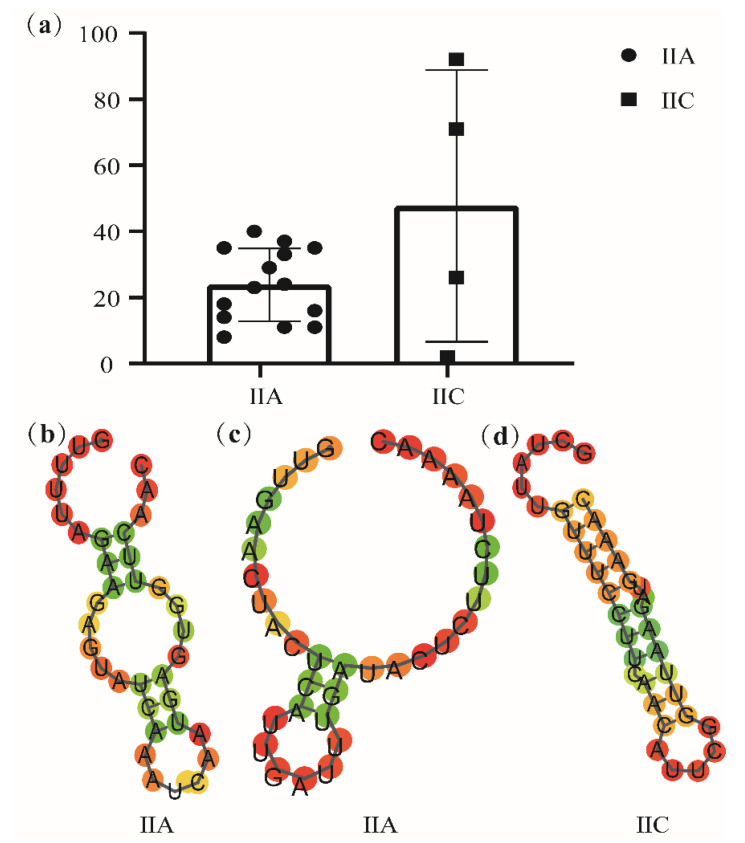
Prediction of CRISPR–Cas systems in *Latilactobacillus sakei*. (**a**) Number of spacer sequences of CRISPR loci in different subtypes of *Latilactobacillus sakei*; (**b**–**d**) Predicted RNA secondary structures of CRISPR DR in *Latilactobacillus sakei*.

**Figure 6:**
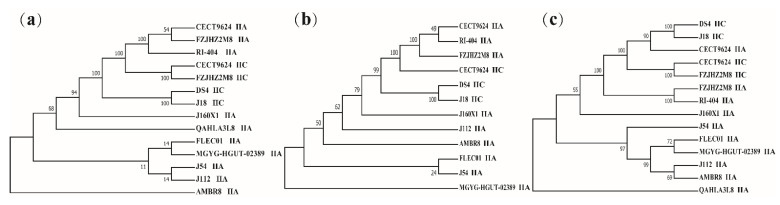
CRISPR–Cas phylogenetic analyses for *Latilactobacillus. sakei*. (**a**) Phylogenetic tree based on the Cas1 protein; (**b**) Phylogenetic tree based on the Cas2 protein; (**c**) Phylogenetic tree based on the Cas9 protein.

By analyzing the number of spacer sequences of CRISPR loci with different subtypes of *L. sakei* (Figure 5a), it is clear that the number of interval sequences of type IIC loci is quite different, up to 92 and at least 2. Repeat sequences were explored by their secondary structure (Figure 5b–d). According to the repeated sequences, it is predicted that there are two typical secondary structures in subtype IIA (Figure 5b–c) and one typical secondary structure in subtype IIC (Figure 5d). Repeated sequence is a typical stem–loop stable structure, which contains a large loop and a small loop at both ends. The phylogenetic tree was constructed with Cas1, Cas2, and Cas9 protein genes, showing that Cas1, Cas2, and Cas9 protein genes of subtype IIA and IIC have strict single-line inheritance, but there were a few exceptions (Figure 6a–c).

## 4. Discussion

*Latilactobacillus sakei* is a potential candidate probiotic that exists in many niches. A study showed that *L. sakei* can not only be used as a starter [56] and biological protective agent [57] in the food industry, but also has a variety of probiotic functions, such as improving metabolic syndromes such as obesity [58], improving immunity and relieving atopic dermatitis [59], and alleviating inflammatory reactions in colitis mice [60]. The development of genomic tools provides strong support for diversity analysis of strains. However, there are few studies on the genome diversity of *L. sakei*, and the isolation sources of strains are relatively single. In this study, the genetic diversity and functional diversity of 68 *L. sakei* strains from different niches were analyzed using 14 *L. sakei* strains isolated in our laboratory and 54 strains with published genetic information on NCBI.

The average genome size of 68 strains of *L. sakei* was 1.98 Mb, and the average GC content was 41.22%, which is consistent with the study of Eisenbach et al. [61]. The average GC content is lower than that of *Ligilactobacillus ruminis* [62], *Lacticaseibacillus casei* [63], and other lactobacillus strains that belong to a free-living and nomadic lifestyle, although higher than that of *Pediococcus pentosaceus* [64] and *Lactobacillus crispatus* [65]. The pan-genome and core genome showed that *L. sakei* has an open pan-genome. In addition, the function and translation, defense mechanism, and general function prediction of *L. sakei* were revealed by annotating the core genes.

ANI is a classical index, used to distinguish whether a particular strain belongs to the same species, and commonly takes a 95% threshold as the species boundary [55]. Except for *L. sakei* DS4, the ANI value of other strains was more than 97%, whereas that of DS4 was only 92%, which reveals that DS4 may be a potential subspecies. Based on the phylogenetic tree of homologous gene sequence similarity, 68 *L. sakei* strains were divided into two major clades and three main sub-clades. There was no evident correlation between each branch and the isolated source.

To further understand the fermentation ability of *L. sakei* using carbohydrates, the genome of 14 strains of *L. sakei* isolated in our laboratory was analyzed and compared using CAZymes technology. GH family enzymes, including GH1, GH2, GH42, and GH73, are the relatively abundant carbohydrate enzymes in *L. sakei*. These enzymes are involved in the catabolism of various carbon compounds, such as soluble starch, mannose, xylose, glucose, trehalose, maltose, and galactose, consistent with the results of Eisenbach et al. [61]. Aside from the corresponding hydrolases, the phosphotransferase system will also affect the utilization of carbohydrates. Chaillou et al. [20] showed that there are phosphotransferase systems of glucose, mannose, fructose, sucrose, and trehalose in *L. sakei*, which provides a stronger basis for utilizing these carbohydrates by *L. sakei.* GH25, as lysozyme [66], participates in peptidoglycan and cell wall catabolism, which can promote cell division and defense. β-N-acetylglucosamine is encoded by GH73, and the β-1,4 glycosidic bond between N-acetylmuramic acid and N-acetylglucosamine of bacterial cell wall peptidoglycan is cleaved by it [67]. These results provide a competitive advantage for the survival of *L. sakei*. Microbes also produce a special enzyme called carbohydrate esterase (CE) to deacetylate hemicellulose and pectin units of plant polysaccharides [68]. CE is mainly divided into pectin deacetylating CE and hemicellulose deacetylation. *L. sakei* is rich in the CE1 family, which is an acetyl xylan esterase.

Owing to their intrinsic and nontransmissible characteristics, many LABs have high antibiotic susceptibility [69]. All 14 strains of *L sakei* isolated in our study have glycopeptide resistance genes; therefore, they have good tolerance to vancomycin, a typical antibiotic of glycopeptide. This is consistent with the results of Georgieva et al. [70], who found that heterofermentative lactic acid bacteria are resistant to vancomycin. In addition, 14 *L. sakei* strains have glycoside resistance genes, which are resistant to streptomycin, kanamycin, and gentamicin, which are inherent properties of lactobacillus [71]. There were 13 tetracycline resistance genes in the studied *L. sakei*, however, six strains were sensitive to tetracycline, among which four strains were isolated from fecal samples. The resistance of most bacteria to tetracycline seems to be obtained horizontally, and the *tet* gene encoding drug resistance is highly mobile because it is located on conjugated transposons [72]. Most *tet* genes encode tetracycline resistance efflux proteins, a part of the major facilitator (MFS) of transporters. These proteins are bound by membranes and exchange protons with tetracycline cation complexes under concentration gradient [73], which reduces the concentration of tetracycline in cells, thus protecting ribosomes in cells.

CRISPR and Cas combine to form the CRISPR–Cas system, providing adaptive immunity to bacterial invasive components [74]. Sixteen strains of 68 *L. sakei* strains were identified to have a complete CRISPR–Cas system, including 14 subtypes of IIA and 4 subtypes of IIC. This is consistent with the results of Ilkkan et al. [30] that subtype IIA is the main CRISPR–Cas subtype in *L. sakei*. In *Latilactobacillus curvatus*, *Loigolactobacillus rennini*, *Pediococcus damnosus,* and *Secundilactobacillus paracollinoides*, which exist in a similar niche to *L. sakei*, highly similar structures of type IIA CRISPR/Cas and similar Cas protein sequences were also found [33]. The activity of the CRISPR system is reflected by the number of spacer sequences, and continuous acquisition of spacer sequences has been proven in the active CRISPR–Cas system [75]. It can be inferred from the number of spacer sequences that the IIC subtype of *L. sakei* is more active and can better resist the insertion of foreign genes. The CRISPR–Cas structure can be used in genetic engineering, and its great potential has been proven, particularly Cas9 nuclease [76]. Cas protein is used as a programmable nuclease that is used for efficient and accurate genome editing in various fields of medicine, research, and biotechnology. The new possibility of Cas9-mediated genome editing is attributed to the identification of new PAMs(g/a) AAA (for type IIA) and (a/g) (c/t) AC (for type IIC CRISPR–Cas system), as well as localized tracrRNAs [32]. Therefore, the application potential of *L. sakei* Cas nuclease requires further study.

## 5. Conclusions

In this study, comparative genomics was used to analyze the genomes of 68 *Latilactobacillus sakei* strains, which provided a basis for analyzing the functional genes of this species. The results showed that niche affected the antibiotic resistance of *L. sakei*, and the strains from feces and pickle water had more abundant antibiotic resistance genes, and its genetic diversity is also reflected in carbohydrate utilization and some immune/competition related factors (CRISPR). Genome sequencing and genetic analysis in this study helped understand the biotechnology potential of *L. sakei* and promote its future development as a protective agent/starter and a therapeutic agent for microbial-related diseases.

## Figures and Tables

**Table 1:** The information of 68 *Latilactobacillus sakei* strains.

Strain	Source	Genome Size (Mb)	GC (%)	CDS No.	Reference
FAHWH17-L1	Human feces	1.90	41.82	1860	This work
FJLHD1M1	Human feces	1.98	41.81	1969	This work
FJLHD26M6	Human feces	1.98	41.79	1950	This work
FXJWS8M1	Human feces	2.11	41.00	2142	This work
FZJHZ2M8	Human feces	2.18	41.61	2085	This work
GD179	Human feces	1.88	41.83	1856	This work
FLEC01	Human feces	1.96	41.24	1919	[25]
QYNXSBNJH59L1	Sour cowhide	1.94	41.77	1928	This work
QJSSZ1L4	Air-dried sausage	1.90	41.92	1876	This work
QJSNT1L10	Air-dried sausage	1.85	41.86	1829	This work
QAHLA3L8	Air-dried sausage	1.90	42.03	1858	This work
DSM15831	Meat	1.99	41.00	1924	[26]
RI-496	Meat	2.02	40.90	1934	[26]
RI-499	Meat	1.91	41.00	1838	[26]
RI-500	Meat	2.03	40.90	1992	[26]
RI-394	Meat	1.94	41.00	1963	[27]
23K	French sausage	1.88	41.30	1779	[20]
J64	Dry sausage	2.05	41.06	2102	[25]
J156	Dry sausage	1.88	41.07	1817	[25]
J112	Dry sausage	1.98	41.04	1913	[25]
J54	Dry sausage	2.01	41.31	1982	[25]
J18	Dry sausage	1.82	41.00	1826	[25]
MFPB16A1401	Beef carpaccio	2.04	41.09	1960	[25]
MFPB19	Beef carpaccio	2.06	41.05	1977	[25]
J160 × 1	Horse meat	1.90	41.07	1857	[25]
FAM18311	Fermented meat products	1.95	41.3	1896	[28]
JXJ41	Kimchi water	2.04	41.80	2010	This work
RS704	Kimchi water	1.96	41.86	1945	This work
DS4	Kimchi	2.10	41.41	1970	[26]
Probio65	Kimchi	2.08	41.18	1984	[26]
WiKim0063	Kimchi	2.08	41.17	1955	[26]
WiKim0072	Kimchi	2.03	41.17	1936	[26]
WiKim0073	Kimchi	2.04	41.16	1913	[26]
WiKim0074	Kimchi	2.04	41.03	1916	[26]
WiKim22	Kimchi	2.19	40.61	1869	[29]
CBA3614	kimchi	2.02	41.13	1910	[30]
MBEL1397	Kimchi	1.99	41.04	1847	[31]
FBL1	Mukeunji	2.03	41.20	1936	[32]
QJSSZ4L10	Pickled green vegetable heart	2.05	41.70	2075	This work
QJLYJ4L4	Pickled radish	1.97	41.76	1928	This work
CECT9267	Fermented potatos	2.03	40.90	1972	[26]
CECT9624	Fermented potatos	2.06	40.80	1986	[33]
ZFM229	Fermented vegetables	2.02	41.22	1907	[30]
LZ217	Fermented vegetables	2.02	41.22	1908	[30]
RI-516	Cacao bean	1.91	41.00	1847	[26]
RI-517	Cacao bean	1.95	41.00	1878	[26]
LS25	Commercial product Bitec LS-25	2.02	40.90	1972	[34]
DSM20017	Sake	1.91	41.1	1820	[26]
TMW1.411	Starter culture	1.94	41.06	1859	[33]
LK-145	Japanese sake cellar	1.99	41.15	1901	[35]
NBRC3541	Raw moto-shu	1.94	41.10	1855	[36]
NBRC15893	Kimoto	1.90	41.10	1822	[37]
ATCC15521	Moto, starter of sake	1.94	41.10	1835	Unknown
ZFM220	Raw cow milk	2.02	41.22	1909	[30]
ZFM225	Raw cow milk	2.02	41.22	1909	[30]
AMBR8	Nasopharyngeal samples	2.00	41.00	1923	[38]
RI-493	Unknown	1.97	40.90	1890	[26]
CBA3635	Unknown	2.06	41.10	2002	[30]
L15	Unknown	1.98	41.00	1915	[30]
ye2	Unknown	1.99	41.10	1907	[30]
RI-403	Unknown	2.00	41.00	2032	[27]
RI-404	Unknown	1.95	40.90	1977	[27]
RI-409	Unknown	1.99	41.00	2022	[27]
RI-410	Unknown	1.93	41.10	1949	[27]
RI-412	Unknown	1.92	41.10	1934	[27]
MGYG-HGUT-02389	Unknown	1.96	41.20	1813	Unknown
ERR260134-bin.14	Unknown	1.52	41.20	-	Unknown
NBRC107868	Unknown	1.97	41.00	1920	Unknown

“-”: Information missing.

**Table 2:** MIC of different antibiotics for *Latilactobacillus sakei*.

Strain	Concentration (μg/mL)										
	RD	S	K	CN	TE	DA	N	E	CIP	VA	C
FJLHD1M1	1	256	256	1024	2	0.5	256	1	128	128	8
GD17-9	1	256	128	1024	2	0.5	256	1	64	128	8
FAHWH17-L1	0.5	512	256	1024	16	2	256	4	128	128	16
JXJ4-1	1	512	128	1024	64	0.5	64	4	128	128	128
QYNXSBNJH59L1	0.25	512	256	1024	4	0.5	256	2	128	128	32
QJSSZ4L10	0.5	512	512	1024	8	0.25	128	4	128	128	32
QJLYJ4L4	0.5	256	128	256	0	0.25	256	8	128	128	16
QJSSZ1L4	1	512	256	1024	32	0.5	256	1	128	128	16
QAHLA3L8	1	256	256	1024	32	0.5	256	2	64	128	32
FJLHD26M6	0.25	256	256	512	4	0.125	32	2	128	128	8
FXJWS8M1	8	256	256	1024	2	0.25	128	1	64	128	8
FZJHZ2M8	1	256	512	256	64	4	256	2	128	128	16
RS704	2	256	128	512	64	0.5	256	2	64	128	8
QJSNT1L10	0.125	128	128	1024	64	0.0625	128	0.5	32	128	128

RD: rifampicin; S: streptomycin; K: kanamycin; CN: gentamicin; TE: tetracycline; DA: clindamycin; N: neomycin; E: erythromycin; CIP: ciprofloxacin; VA: vancomycin.; C: chloramphenicol.

**Table 3:** CRISPR–Cas systems in *Latilactobacillus sakei*.

Strains	Repeat Sequence (5’-3’)	CAS_ Subtype	Repeat Length	No. Spacer	Cas1	Cas2	Cas9	Csn2
FZJHZ2M8	GTTTTAGAAGAGTATCAAATCAATGAGTAGTTCAAC	IIA	36	16	Y	Y	Y	Y
	ATTTCATCTTAACCGAATGTTGAAGGAAACAATAGC	IIC	36	71	Y	N	Y	N
QJSNT1L10	GTTTTAGAAGAGTATCAAATCAATGAGTAGTTCAAC	IIA	36	18	N	N	N	Y
QAHLA3L8	GTTGAACTACTCATTGATTTGATACTCTTCTAAAAC	IIA	36	33	Y	N	Y	Y
DS4	GCTATTGTTTCCTTCAACATTCGGTTAAGATGAAAT	IIC	36	26	Y	Y	Y	N
J18	GCTATTGTTTCCTTCAACATTCGGTTAAGATGAAAC	IIC	36	92	Y	Y	Y	N
CECT9624	GTTTTAGAAGAGTATCAAATCAATGAGTAGTTCAAC	IIA	36	8	Y	Y	Y	Y
	ATTTCATCTTAACCGAATGTTGAAGGAAACA	IIC	31	2	Y	Y	Y	N
J54	GTTGAACTACTCATTGATTTGATACTCTTCTAAAAC	IIA	36	29	Y	Y	Y	Y
J112	GTTTTAGAAGAGTATCAAATCAATGAGTAGTTCAAC	IIA	36	14	Y	Y	Y	N
FLEC01	GTTGAACTACTCATTGATTTGATACTCTTCTAAAAC	IIA	36	35	Y	Y	Y	Y
J160 × 1	GTTGAACTACTCATTGATTTGATACTCTTCTAAAAC	IIA	36	24	Y	Y	Y	Y
MGYG-HGUT-02389	GTTGAACTACTCATTGATTTGATACTCTTCTAAAAC	IIA	36	35	Y	Y	Y	Y
DSM15831	AGTTGAACCACTCATTGATTTGATACTCTTCTAAAAC	IIA	37	11	N	N	N	Y
RI-403	GTTTTAGAAGAGTATCAAATCAATGAGTGGTTCAAC	IIA	36	40	N	N	N	Y
RI-404	GTTTTAGAAGAGTATCAAATCAATGAGTGGTTCAAC	IIA	36	37	Y	Y	Y	Y
AMBR8	GTTGAACCACTCATTGATTTGATACTCTTCTAAAAC	IIA	36	23	Y	Y	Y	Y
NBRC107868	GTTTTAGAAGAGTATCAAATCAATGAGTGGTTCAACT	IIA	37	11	N	N	N	Y

Y: present; N: none.

## Data Availability

Not applicable.
